# Worsening hearing was associated with higher β-amyloid and tau burden in age-related hearing loss

**DOI:** 10.1038/s41598-022-14466-6

**Published:** 2022-06-21

**Authors:** Mengmeng Zheng, Jiangyu Yan, Wenjuan Hao, Yuan Ren, Ming Zhou, Yunzhi Wang, Kai Wang

**Affiliations:** 1 Department of Otorhinolaryngology Head and Neck Surgery, Hwa Mei Hospital, University of Chinese Academy of Sciences, 41 Xibei street, Ningbo, 315010 Zhejiang China; 2 Department of Otorhinolaryngology Head and Neck Surgery, Ningbo Medical Center Lihuili Hospital, Ningbo, 315000 China; 3 Department of Clinical Medicine, School of Medicine, Ningbo University, Ningbo, China; 4 School of Health Sciences, University of Sydney, Sydney, NSW 2050 Australia

**Keywords:** Cognitive ageing, Learning and memory

## Abstract

Age-related hearing loss (ARHL) represents the frequently occurring disability that affects the elderly worldwide. The recent evidence has calculated ARHL to be most potential risk factor to predict dementia. β-amyloid plaques and tau accumulation in brain are hallmarks pathologic feature of Alzheimer’s disease (AD), which is a leading cause resulting in dementia. However, the potential mechanistic associations between ARHL and dementia remains unknown. We performed the present cross-sectional cohort study by enrolling 72 patients from research on hearing as well as the pathologic hallmarks of AD in brain. The exposure of hearing was measured by either word recognition score or mean pure-tone of the superior ear. The brain β-amyloid and tau standardized uptake value ratio (SUVR) were measured by positron emission tomography (PET). The covariates included gender, age, cardiovascular disease, education and hearing aid use. To analyze the association between hearing and β-amyloid/tau, linear regression was used and adjusted for potentially confounding covariates. Our data showed that the mean age was 67.1 ± 2.9 years. After adjusted for all the covariates, SUVR of β-amyloid showed an increase of 0.028 [95% confidence interval (CI) 0.004–0.061; *P* = 0.026], while that of tau exhibited an increase of 0.026 (95% CI 0.003–0.056; *P* = 0.033) per mean pure-tone increase by 10 dB (worsening). Likewise, per mean word-recognition score increase by 10%, the SUVR of β-amyloid showed an increase of 0.060 (95% CI 0.008–0.113; *P* = 0.023), while that of tau exhibited an increase of 0.059 (95% CI 0.009–0.111; *P* = 0.031). Taken together, our data demonstrates that hearing worsening was related to the increased burdens of β-amyloid as well as tau detected by PET, which were the AD pathological markers.

## Introduction

Presbycusis or age-related hearing loss (ARHL) refers to hearing loss, which impacts nearly 2/3 elderly people aged > 70 years^1,2^. The ARHL impairs more than sensory situations as it is difficult for people with ARHL to understand and process speech. Greater efforts should be made for compensating auditory input reduction, and this may show adverse outcomes^3^. Recent evidence from a prominent study determines ARHL to be the only most potential risk factor that predicts dementia^4,5^. The recent longitudinal studies support this relationship between ARHL and cognitive decline^6,7^.

Dementia causes great burden on public health globally, and it is frequently caused by Alzheimer’s disease^8,9^ (AD). Countless studies have proved that the elevated level of β-amyloid plaques and tau accumulation in brain are hallmarks pathologic feature of AD^10–12^. Several studies have linked ARHL specifically to AD^13,14^. The theoretical model suggested that ARHL may change the brain structure and lead to AD^2,15^. The magnetic resonance imaging (MRI) also showed that ARHL patients are associated with reduced whole-brain and regional volumes^16,17^. The longitudinal and cross-sectional research adjusted for possible confounding factors also links the ARHL to AD^18^. There are rare studies associating ARHL with specific AD biomarkers.

Positron emission tomography (PET) for β-amyloid and tau made it possible to noninvasively measure the β-amyloid plaques and tau accumulation in brain^19–21^. In this study, we design the novel cohort study for clarifying the possible relation of ARHL with dementia. This study explored the relation of audiometric ARHL with typical tau and β-amyloid burdens in AD, thus clarifying the relation of ARHL with AD.

## Materials and methods

### Participants in this study cohort

Participants were recruited through Ningbo Medical Center Lihuili Hospital from February 2018 to October 2021. Inclusion criteria of this work were as follows, patients aged 52–74 years, being able to receive neuropsychological assessments, PET/MRI examinations, phlebotomy, and no open wounds surrounding auricle. Patients conforming to the following criteria were excluded, existing neurological or psychiatric diseases. All subjects were right-handed. Ethics Committee of Ningbo Medical Center Lihuili Hospital approved this work. All participants were paid for participation and provided the informed consent. Our work described has been carried out in accordance with The Code of Ethics of the World Medical Association (Declaration of Helsinki) for experiments involving humans.

### Hearing (exposure)

Prior to hearing test, patients were inquired of ear disease history, hearing device wearing, ear surgery and perceived ARHL. The subjects with history of common ear diseases include tinnitus, otitis media, tympanic membrane perforation, external auditory canal inflammation were excluded in the hearing test. A clinically validated ipad-based portable audiometer (ShoeBox; Canada) was used to assess hearing. Each participant was in the tranquil room that had < 45 dB environment noise, asked to wear professional sound-attenuating headphones after calibration and remove any hearing device. Later, the thresholds of air-conduction pure-tone were determined. We calculated the mean pure-tone (in dB hearing level) by the mean hearing threshold at 500/1000/2000/4000 Hz. In addition, we detected word recognition by percentage of Chinese words that were repeated accurately and displayed in the suprathreshold loudness (+ 35 dB for PTA < 50 dB, + 30 dB for PTA 50–59 dB, + 20 dB for PTA 60–69 dB, + 10 dB for PTA ≥ 70 dB). Just immediate word repeating was required during word recognition test, with no requirement of memory-based word recalling. The pure-tone average in the better hearing ear was the primary exposure variable and the word recognition score was the secondary exposure variable. Subjects had normal hearing (average of pure-tone thresholds _0.5–4.0 kHz_ ≤ 25 dB) and subjects had HL (average of pure-tone thresholds _0.5–4.0 kHz_ > 25 dB) and the average of pure-tone refers to the mean value in the right and left ears.

### SUVR calculation

For β-amyloid, 18F-florbetaben PET/computed tomography (CT) scans (Siemens, Germany) were used. Images were collected within 20 min from 90 min after injection. For tau, [^18^F]flortaucipir PET/ CT were used. Here, we obtained PET data within 80–100 min following [^18^F]flortaucipir injection. In addition, we registered PET images together with relevant CT scans. Standardized uptake value ratio (SUVR) represents the normalized absorption value in every voxel relative to standard gray matter absorption value in the epencephalon. The burden of β-amyloid served as the dependent variable, which was verified to be global SUVR detected using 18F-florbetaben PET. The lateral temporal cortex, cingulate cortex, parietal cortex and frontal cortex were included in the calculation of the overall mean β-amyloid in separate region of interest (ROI) based on different vortexes. In the sensitivity analysis, high and low levels of β-amyloid were binarized according to median SUVR.

### Covariates

This study enrolled covariates like age (years), sex (female, male), education (years), wearing of hearing device (yes/no) and cardiovascular disease (CVD). We determined CVD as the composite score (range, 0–4), and added 1 point to every condition below, diabetes, stroke, heart attack and hypertension (blood pressure BP > 140/90). This composite score was created to avoid multicollinearity in regression modeling. If only one of the four individual variables were missing, the missing value was imputed by taking the mean of the other three scores.

### Statistical analysis

Relation of ARHL with tau/β-amyloid SUVR was analyzed by multiple linear regression, controlling for covariates. Relation of ARHL with binary tau/β-amyloid SUVR was analyzed by multivariable logistic regression in the sensitivity analysis. Except as otherwise noted, data were displayed as mean ± SD. RStudio 1.2 in R programming language v3.6.2 was utilized for data analysis.

## Results

### Description of the enrollment and inclusion

**Figure 1:**
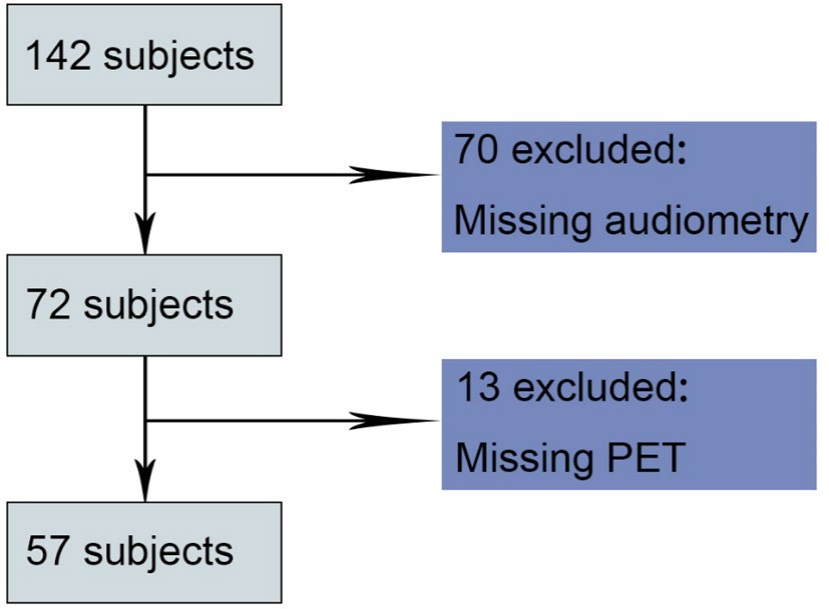
Diagram showing criteria to include and exclude participants. PET, positron emission tomography.

The current study included 142 subjects and 70 subjects withdrew from the study because of the COVID-19. 72 subjects (aged 52–74 years) had a hearing test and 57 subjects (aged 64–70 years) had collected β-amyloid and tau PET (see “Materials and methods” section), which comprised the analytic sample (Fig. 1).

### The cohort characteristics

**Figure 2:**
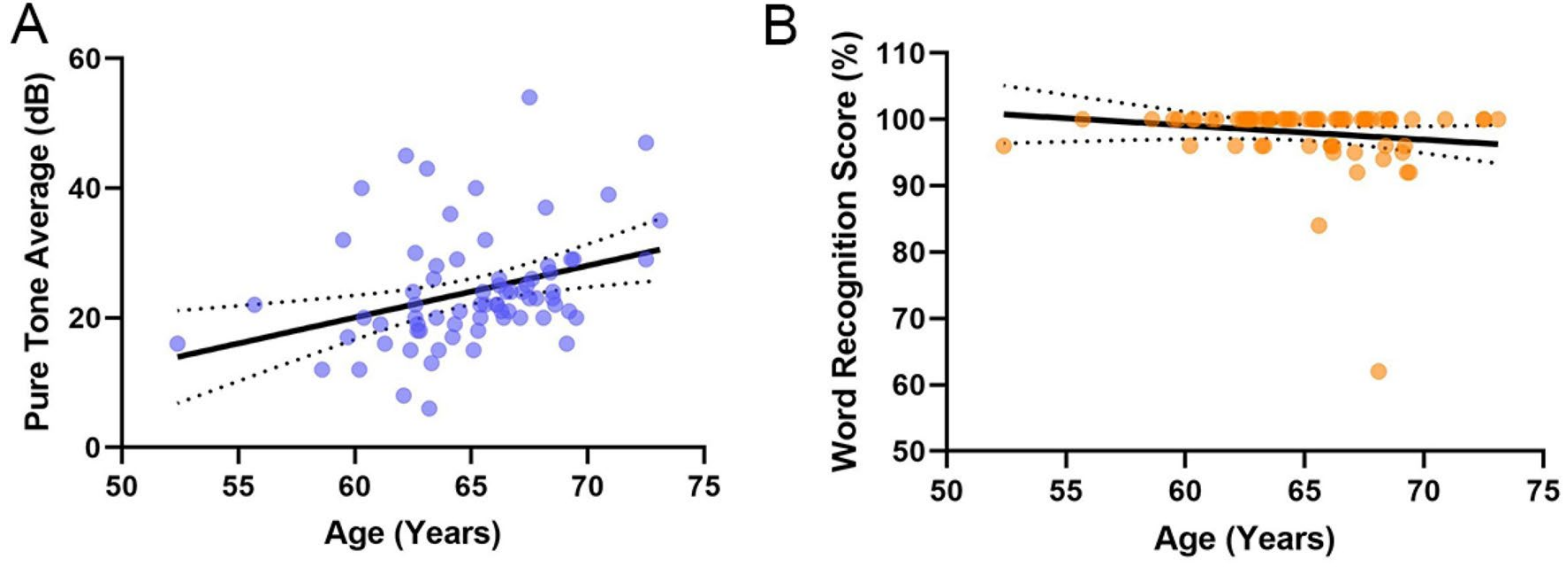
Scatterplots showing age and hearing distribution and their relation. (**A**) Mean pure-tone (**A**) and word-recognition score (**B**) versus age.

The demographics and characteristics of subjects in PET scan were presented in Table 1. The cohort was 62.5% women. 65.3% subjects had normal hearing and 34.7% of subjects had HL, an expected proportion given the community-based sample. 2.8% of subjects had hearing aid use and no one had implanted hearing devices. The pure-tone average in normal hearing group was 15.8 dB (± 4.3 dB), with a range of 4–24.8 dB. In contrast, the pure-tone average in ARHL group was 34.2 dB (± 5.7 dB), with a range of 25.2–56 dB. The overall word recognition score was 97.9 ± 4.8, with a range of 60–100% (Table S1). The overall β-amyloid level was 1.24 (± 0.17). Figure 2 showed the distribution and relationship of age and hearing variables. The cohort in this study was middle and late adults, so relatively narrow distribution of ARHL severity and age range were observed.

**Table 1:** The demographic and audiological variables of subjects included in the analyses.

Demographics	Overall	Normal hearing (≤ 25 dB)	Hearing loss (> 25 dB)	*P* value
No.%	72	47(65.3%)	25(34.7%)	N/A
Pure-tone average, mean ± SD	20.1 ± 8.5	15.8 ± 4.3	34.2 ± 5.7	0.007**
Word recognition score, mean ± SD	97.9 ± 4.8	98.7 ± 3.1	94.8 ± 8.7	0.014*
Age, year, mean ± SD	67.1 ± 2.9	66.7 ± 2.5	68.0 ± 2.4	0.17
Women, no. (%)	45(62.5%)	32(71.1%)	13(28.9%)	0.046*
Hearing aid use, no. (%)	2(2.8%)	0(0%)	2(2.8%)	0.043*
Education, year, mean ± SD	10.2 ± 2.8	10.8 ± 2.3	9.1 ± 2.5	0.27
Global tau SUVR, mean ± SD	1.62 ± 1.4	1.61 ± 1.2	1.63 ± 1.3	0.053
Global β-amyloid SUVR, mean ± SD	1.24 ± 0.17	1.21 ± 0.15	1.29 ± 0.12	0.047*

For continuous variables, t tests were indicated, and for categorical variables, χ2 tests or fisher exact were used.

*SD* standard deviation, *N/A* not applicable, *SUVR* standardized uptake value ratio.

*Significant, *P* < 0.05.

### Regression analyses

**Figure 3:**
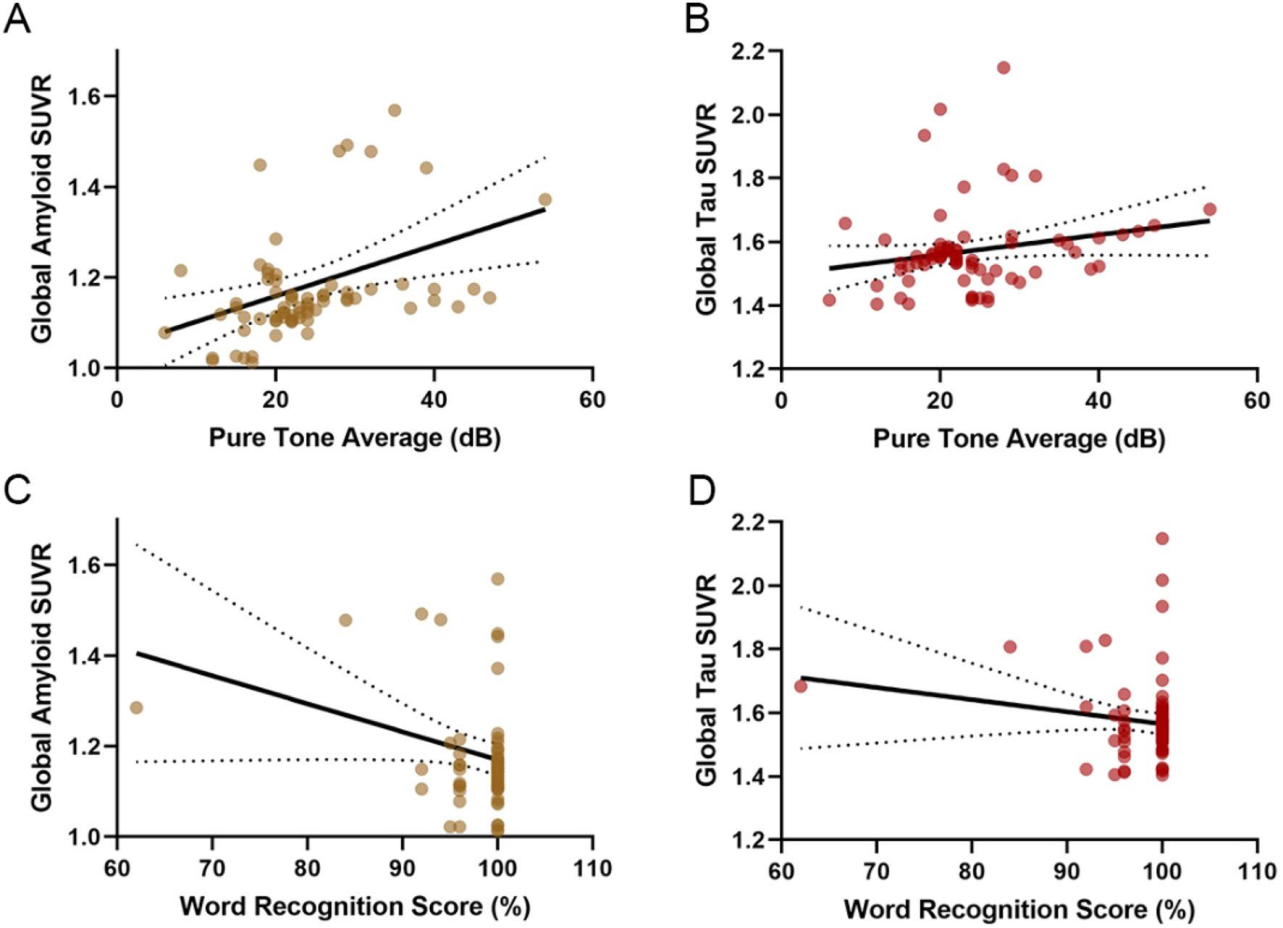
Univariable linear regression models of β-amyloid/tau SUVR based on hearing. (**A**) β-amyloid versus pure-tone average. (**B**) Tau versus pure-tone average. (**C**) β-amyloid versus word recognition score. (**D**) Tau versus word recognition score.

In univariable regression, the mean pure-tone detected hearing was significantly related to SUVR of β-amyloid (*P* = 0.047) (Fig. 3A and Table 2), while a near-significant relationship between pure-tone average and tau SUVR (*P* = 0.053) (Fig. 3B and Table 2). When the covariates such as sex, age, education, CVD as well as wearing of hearing device were controlled in multivariable regression, connection of mean pure-tone-detected hearing with SUVR of tau/β-amyloid became more significant. After adjusting for age and gender, the SUVR of β-amyloid showed an increase of 0.027 (95% CI 0.003–0.054), while that of tau showed an increase of 0.024 (95% CI 0.002–0.053) per 10 dB mean pure-tone elevation (worsening). When we incorporated age, gender, education and CVD into the model, relation between pure-tone average and β-amyloid/tau SUVR were strengthened. When all the covariates were added to the model, including hearing aid use, education, cardiovascular disease, age and gender, the relationship between pure-tone average and β-amyloid/tau SUVR were slightly attenuated. In this model, the β-amyloid SUVR increased by 0.028 [95% confidence interval (CI) 0.004–0.061; *P* = 0.026] and tau SUVR increased by 0.026 [95% confidence interval (CI): 0.003–0.056; *P* = 0.033] for every 10 dB increase (worsening) in pure-tone average on average (Table 2).

**Table 2:** Regression models for global β-amyloid and Tau SUVR based on pure-tone average hearing.

Model	Global β- Amyloid SUVR difference per 10 dB worsening in pure-tone average (95% CI)	*P*	Global Tau SUVR difference per 10 db worsening in pure-tone average (95% CI)	*P*
1. Univariable: pure-tone average	0.023 (0.00–0.051)	0.047	0.022 (0.00–0.048)	0.053
2. Multivariable (model 1 + age, gender)	0.027 (0.003–0.054)	0.028*	0.024 (0.002–0.053)	0.041*
3. Multivariable (model 2 + education, cardiovascular disease)	0.031 (0.004–0.059)	0.012*	0.029 (0.006–0.058)	0.029*
4. Multivariable (model 3 + hearing aid use) [fully adjusted model]	0.028 (0.004–0.061)	0.026*	0.026 (0.003–0.056)	0.033*

*SUVR* standardized uptake value ratio, *CI* confidence interval.

*Significant, *P* < 0.05.

In univariable regression, the word-recognition score-determined hearing was significantly related to tau/β-amyloid SUVR. To be specific, per 10% mean word-recognition score decrease (worsening), the SUVR of β-amyloid showed an increase of 0.0054 (95% CI 0.016–0.100; *P* = 0.007) (Fig. 3C and Table 3) and that of tau showed an increase of 0.052 (95% CI 0.014–0.009; *P* = 0.005) (Fig. 3D and Table 3). When the covariates such as sex, age, CVD, wearing of hearing device and education were incorporated into multivariate analysis, word-recognition score was still related to SUVR of tau/β-amyloid. When all the covariates were added to the model, including hearing aid use, education, cardiovascular disease, age and gender, per 10% mean word-recognition score decrease, the SUVR of β-amyloid showed an increase of 0.060 (95% CI 0.008–0.113; *P* = 0.023) while that of tau showed an increase of 0.059 (95% CI 0.009–0.111; *P* = 0.031) (Table 3).

**Table 3:** Regression models for GLOBAL β-amyloid and Tau SUVR based on word recognition score hearing.

Model	Global β-amyloid SUVR difference per 10 dB worsening in word recognition score (95% CI)	P	Global Tau SUVR difference per 10 dB worsening in word recognition score (95% CI)	*P*
1. Univariable: pure-tone average	0.054 (0.016–0.100)	0.007**	0.052 (0.014–0.009)	0.005**
2. Multivariable (model 1 + age, gender)	0.063 (0.018–0.104)	0.004**	0.059 (0.016–0.101)	0.004**
3. Multivariable (model 2 + education, cardiovascular disease)	0.064 (0.018–0.106)	0.006**	0.062 (0.019–0.104)	0.009**
4. Multivariable (model 3 + hearing aid use) [fully adjusted model]	0.060 (0.008–0.113)	0.023*	0.059 (0.009–0.111)	0.031*

*SUVR* standardized uptake value ratio, *CI* confidence interval.

*Significant, *P* < 0.05.

We later conducted binary regression on the SUVR of β-amyloid (high and low classified according to the median 1.22 ± 0.14) as well as tau (high and low, classified according to the median 1.60 ± 0.12) in the sensitivity analysis, as completely adjusted model outcomes. Per 10 dB mean pure-tone increase, the SUVR of β-amyloid showed an increase of 2.09-fold, while that of tau showed an increase of 1.97-fold. No significance was observed between word recognition score and binary β-amyloid/tau SUVR and there were no influential outliers on regression diagnostics.

## Discussion

In our cross-sectional study, audiometric ARHL has significant relationship with β-amyloid and tau measured on PET scan. The above relation was good for adjusting several covariates, such as CVD, education and hearing aid use. Furthermore, it also helped to measure hearing, either via word-recognition score or mean pure-tone. It is widely accepted that β-amyloid and tau accumulation are hallmarks pathologic feature of AD^22,23^, this study posed the question regarding the mechanical relation between ARHL and AD. The present work has first displayed the relation between ARHL and the two hallmarks pathologic feature of AD.

Growing evidence suggests that ARHL is related to cognitive impairment and dementia^24,25^, and its underlying mechanism should be explored. There are 3 mechanistic pathways put forward, including a confounding, causal pathway, and reverse causal pathway, which may be closely interlinked^26,27^. Firstly, ARHL would lead to social isolation and this in turn may reduce cognitive stimulating input and lead to dementia. Secondly, ARHL may induce cognitive load. Because most efforts, which are normally used to create working memories, are instead diverted to decoding speech. Finally, ARHL may lead to unpredictable changes in brain structure through its efferent connections. The accelerated volume declines in whole brain have been observed in individuals with audiometric ARHL. Since ARHL may increase the long-term risk of dementia, the participants in our study may also have cognitive decline.

A recent study on brain autopsies suggested that ARHL was not related to AD neuropathologic results^28^. Such discrepancy may be because that the objective hearing measure is lacking in their work, which has limited sensitivity of the study. As suggested by Wei Xu et al.^29^, ARHL was related to tau in cerebrospinal fluid (CSF) using MRI, which supported our research. Similarly, one research adopts the distinct ^18^F-florbetapir in PET among the elderly but discovers that ARHL is not related to β-amyloid, but the cause of such discrepancy remains unknown^30^.

There were significant associations between hearing, whether by mean pure-tone or word recognition score, and amyloid/tau SUVR. When included word recognition score as a covariate in the Table 2 model. The mean pure-tone detected hearing was significantly related to SUVR of β-amyloid (*P* = 0.047) and a near-significant relationship between pure-tone average and tau SUVR (*P* = 0.053), When the covariate word recognition score was controlled in multivariable regression, connection of mean pure-tone-detected hearing with SUVR of tau/β-amyloid (*P* = 0.056; *P* = 0.068) became less significant. While included mean pure-tone as a covariate in the Table 3 model. The word-recognition score-determined hearing was significantly related to tau/β-amyloid SUVR, When the covariate mean pure-tone was controlled in multivariable regression, connection of word-recognition score with SUVR of tau/β-amyloid (*P* = 0.12; *P* = 0.37) became less significant.

Certain limitations should be noted. Since small effect size of SUVRs changes were observed, larger scale data sets should be examined to further explore the relation between ARHL and the SUVR change of two hallmarks pathologic feature of AD. Just insufficient data were available in the present longitudinal study, making it impossible to conduct the causal inference or cross-sectional research. The population in this study were largely from southeast China, and the generalizability of our cohort remains to be further verified. Therefore, further research is needed to replicate our findings. In our study, most participants showed normal hearing, and they stood for community-based middle-aged population. However, the relations of ARHL with cognitive impairment and brain volumes in such age population are discovered in previous studies. This opposes the necessity of oversampling ARHL subjects. As moderate-severe ARHL patients are lacking, it is difficult to extend our results to this group. This study has strengths. Our research is the first to show the association between ARHL and the two hallmarks pathologic feature of AD. The binary and continuous results were examined, meanwhile, 2 distinct hearing measuring approaches were adopted. As a result, hearing worsening related to the increased burdens of β-amyloid and tau.

## Supplementary Information

Supplementary Information.

## Data Availability

The datasets used and/or analysed during the current study available from the corresponding author on reasonable request.
