# Supplementary material for: Worsening hearing was associated with higher β-amyloid and tau burden in age-related hearing loss
Source: Sci Rep. 2022 Jun 21;12:10493. doi: 10.1038/s41598-022-14466-6 (PMC9212197; doi:10.1038/s41598-022-14466-6)
Supplement: Supplementary file 1 — Supplementary Information. [file 41598_2022_14466_MOESM1_ESM.docx]

**Supplemental Table S1**. Scattergram of Hearing Levels.

|  |  | | **Word Recognition Score (%)** | | | | | | | | | |
| --- | --- | --- | --- | --- | --- | --- | --- | --- | --- | --- | --- | --- |
| **Pure-Tone Average (dB)** |  | 100 - 90 | | 89 - 80 | 79 - 70 | 69 - 60 | 59 - 50 | 49 - 40 | 39 - 30 | 29 - 20 | 19 - 10 | 9 -  0 |
|  | 0 - 10 | 9 | |  |  |  |  |  |  |  |  |  |
|  | 11 - 20 | 27 | |  |  |  |  |  |  |  |  |  |
|  | 21 - 30 | 22 | |  |  |  |  |  |  |  |  |  |
|  | 31 - 40 | 7 | | 2 |  |  |  |  |  |  |  |  |
|  | 41 - 50 |  | | 1 | 2 | 1 |  |  |  |  |  |  |
|  | 51 - 60 |  | | 1 |  |  |  |  |  |  |  |  |
|  | 61 - 70 |  | |  |  |  |  |  |  |  |  |  |
|  | 71 - 80 |  | |  |  |  |  |  |  |  |  |  |
|  | 81 - 90 |  | |  |  |  |  |  |  |  |  |  |
|  | >91 |  | |  |  |  |  |  |  |  |  |  |
